# The Efficacy of Written Information Intervention in Reduction of Hospital Re-admission Cost in Patients With Heart Failure; A Systematic Review and Meta-Analysis

**DOI:** 10.15171/jcvtr.2015.01

**Published:** 2015-03-29

**Authors:** Vahideh Zarea Gavgani, Faranak Kazemi Majd, Shirin Nosratnejad, Ali Golmohammadi, Homayoun Sadeghi-Bazargani

**Affiliations:** ^1^ Tabriz Health Services Management Research Center, National public Health Management Center (NPMC), and Department of Medical Library and Information Sciences, Tabriz University of Medical Sciences, Tabriz, Iran; ^2^ Faculty of Management and Health Informatics, Tabriz University of Medical Sciences, Tabriz, Iran; ^3^ Iranian Center of Excellence in Health Service Management, Tabriz University of Medical Sciences, Tabriz, Iran; ^4^ Department of Cardiology, Tabriz University of Medical Sciences, Tabriz, Iran; ^5^ Road Traffic Injury Research Centre, Department of Statistical and Epidemiology, Tabriz University of Medical Sciences, Tabriz, Iran

**Keywords:** Heart Failure, Patient Admission, Meta-Analysis

## Abstract

***Objective:*** To assess the efficacy of written information versus non written information intervention in reducing hospital readmission cost, if prescribed or presented to the patients with HF.

***Methods:*** The study was a systematic review and meta-analysis. We searched Medline (Ovid) and Cochrane library during the past 20 years from 1993 to 2013. We also conducted a manual search through Google Scholar and a direct search in the group of related journals in Black Well and Science Direct trough their websites. Two reviewers appraised the identified studies, and meta-analysis was done to estimate the mean saving cost of patient readmission. All the included studies must have been done by randomization to be eligible for study.

***Result:*** We assessed the full-texts 3 out of 65 studies with 754 patients and average age of 74.33. The mean of estimated saving readmission cost in intervention group versus control group was US $2751 (95% CI: 2708 – 2794) and the mean of total saving cost in intervention group versus control group was US $2047 (base year 2010) with (95% CI: 2004 – 2089). No publication bias was found by testing the heterogeneity of studies.

***Conclusion:*** One of the effective factors in minimizing the healthcare cost and preventing from hospital re-admission is providing the patients with information prescription in a written format. It is suggested that hospital management, Medicare organizations, policy makers and individual physicians consider the prescription of appropriate medical information as the indispensable part of patient’s care process.

## Introduction

Heart failure (HF) is a common and serious public health problem and the leading cause of hospitalization among the elderly in developed countries.^1,2^ It imposes a considerable economic burden on the society around the world.^3,4^ Recent assessments suggest a prevalence of congestive HF of 2.3% in Europe.^5^ The estimated total National Health Service (NHS) cost associated with HF in 2000 was £905 million—that is,1.9% of NHS expenditure.^6^ Despite progress in HF treatments, such as increasing use of angiotensin converting enzyme (ACE) inhibitors and more recently b blockade, many agree that the overall management of HF can be improved.^7,8^ Most of the previous reviews of HF disease management programs suggested that specialized follow up of patients by a multidisciplinary team can reduce hospitalization rate.^9,10^ The objective of this systematic review was to determine the impact of written information intervention on hospital re-admission and hospital cost in patients with HF versus to oral information. It was assumed that written information intervention may reduce the frequency and cost of hospital re-admission in HF patients.

The main outcome measure of study was the efficacy of written information intervention on total saving cost of the hospital re-admission in patients with HF.

## Methods

### Study Design

A systematic review and meta-analysis of randomized controlled clinical trial studies.

### Inclusion Criteria

We included randomized controlled clinical trial studies, with primary outcomes of reducing the cost of hospital re-admission after written health information intervention in HF. All the studies with non-randomized control design, non-written information intervention were excluded. We also included the information intervention types such as brochure, health literacy, information prescription, information therapy, patient education and pamphlet and excluded the term ‘bibliotherapy’ in the literature search to cover all formats of written information.

### Search Strategy

A very sensitive strategy was pursued, appropriate to each database, to extract comprehensive and relevant results. Search was performed by a search expert librarian in MEDLINE (Ovid) and Cochrane library from 1993 to 2013, and a manual search was applied using Google Scholar and a direct search in the website of related journals i.e. Black Well and Science Direct to cover the bibliographies of the selected articles from 16 January to 25 February 2013. Language limitation was not considered in searching. The following textual terms with equivalent MeSH headings were used according to PICO.

Patient: Cardiac failure or heart failure patients

Intervention: Information therapy, information prescription, written information intervention

Comparison: Non written information/education intervention

Outcomes: Hospital re-admission, cost, expenditure

### Review Method

Two authors (F.K. and V.G) systematically reviewed the eligible papers through three phases, (I) reading the title and abstract to assess the tentative eligibility, (II) reading the full text to interpret and select the final eligible papers, (III) reviewing the interpretation dispute about the inclusion of studies with vague objectives, methods or reporting. (IV) putting in discussion with the other authors when they face disagreement and uncertainty about the eligibility of a study.

### Selecting the Studies

All identified articles were entered in article management software and the duplicates were eliminated. Then two reviewers (F.K and V.G) independently screened the citations from the literature search for eligibility, titles needed to appear potentially relevant to the study area. Two reviewers independently reviewed and assessed abstracts against three criteria to determine, if (1) the study was a randomized controlled trial; (2) the population of the study was patients with the main diagnosis of HF; (3) that the written information was used as intervention; and (4) at least one of the main outcomes was the cost of re-admission. If the reviewers faced disagreement about the eligibility of a study, they discussed with other authors in a face-to-face discussion session to reach agreement. Full papers were retrieved if both reviewers agreed about the eligibility of papers.

### Quality Appraisal

It was important to ensure that the included studies had been conducted in a way that they met a minimum set of quality criteria. The Critical Appraisal Skills Program (CASP) tool was used for appraising the quality of included studies.

### Data Extraction and Data Sources

One author extracted data from included studies into the data extraction sheet and the other authors checked the extracted data for accuracy and completeness. We focused mainly on the costs as an outcome, the intervention, sample size, the year of study, mean age, and the period of hospitalization, in the selected studies. For each study we collected consumer price index (CPI) and exchanged rate to US $ From the World Bank’s list of indicators.

### Analysis

The reported cost in the included studies was not the same because they had been estimated in various countries and also in various years. We converted all the currencies to US $ using the exchange rate. If it is not reported in the original study, we used the International Monetary Fund (IMF) values (accessed August, 2014). Then the nominal monetary values were corrected using the CPI for the study year (the base year of 2010).

Three studies met our objectives and provided enough data to conduct a meta-analysis. In this meta-analysis we estimated two meta-analyses one for estimating mean cost of readmission in control group and the other for total cost saving by conducting this intervention.

## Result

**Figure 1:**
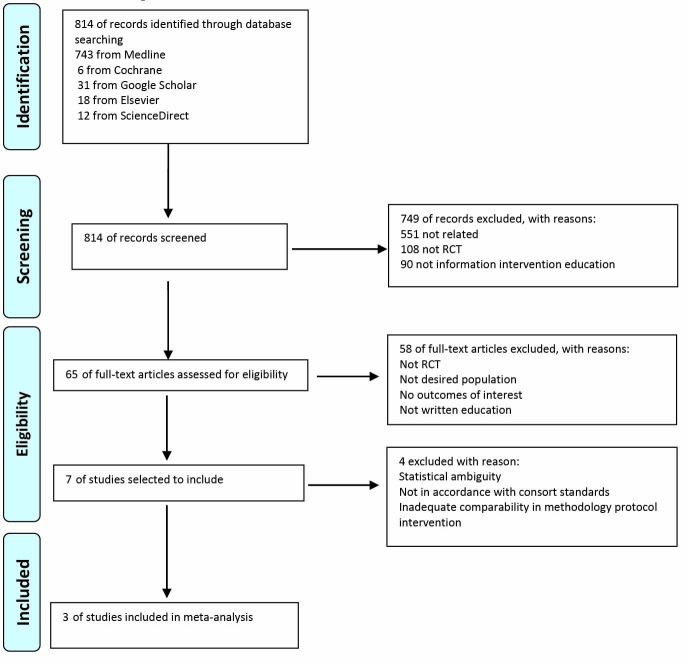
Flow diagram of studies assessed and included in analysis

Our searches yielded 814 hits, 749 articles were excluded after title and abstract screening. We assessed the full text of 65 articles (Figure 1) and finally 3 randomized controlled trials (RCTs)^10-13^ (754 patients) met the eligibility criteria for our analysis. The main interventions in these studies typically involved written information based education on of HF patients.

All 3 studies met suitable quality for being included in our study. Two out of 3 included in studies were performed in Spain and one in the US. The earliest included trial was published in 1995.^13^ Patient inclusion criteria were reasonably similar across all studies. Despite the apparently similar inclusion criteria, the mean age of patients varied between 68–80 years with an average age of 74.33 (Table 1).

**Table 1:** Indicates the Key Features of Included Studies

**Author (Year)**	**Sample** ** Size**	**Location**	**Mean Age**	**Key Components of Intervention**	**Duration of** **Intervention**	**Outcome**
López Cabezas et al (2006)	134	Municipal Hospital of Barcelona, Spain	75	Explaining information with a simple language by audiovisual and written educational material. Frequent telephone follow-up with the objectives of education, counseling. Follow-up visits were Performed at 2, 6 and 12 months after the admissions.	12 months	Re-admission cost in control group = €1575; Re-admission cost in intervention group= € 966; The average cost of the study intervention during the follow-up period per patient = €31; Total saving cost = €578
Atienza et al (2004)	338	Spain	68	Formal education using brochure developed by investigators, explanations of symptoms and signs of heart failure	3 months	Re-admission cost in control group = €5.417; Readmission cost in intervention group = €2.912; The average cost of the study intervention during the follow-up period per patient = €442; Total saving cost = €2063
Rich (1995)	282	Jewish Hospital at Washington University Medical Center, USA	80 in intervention. 78 in control.	Comprehensive education of the patient and family, a prescribed diet, consultation and Intensive follow-up.	3 months	Re-admission cost in control group= $3,236; Re-admission cost in intervention group = $2178; The average cost of the study intervention during the follow-up period per patient = $598; Total saving cost = $460

**Figure 2:**
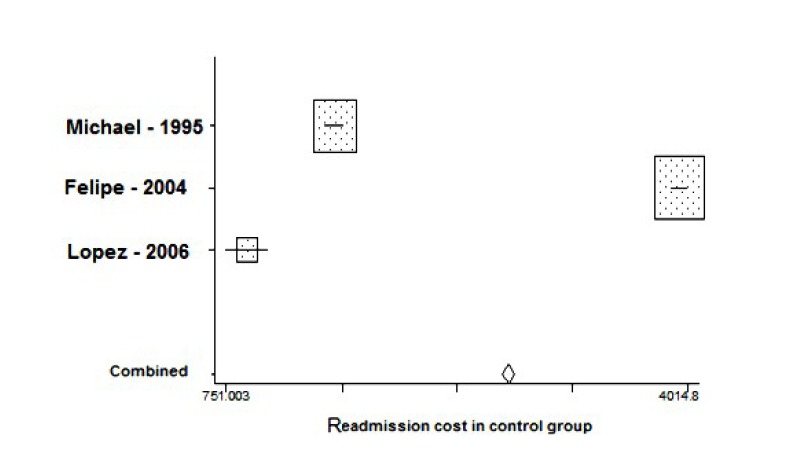
Re-admission cost in control group.

The results of meta-analysis are indicated in Table 2. The mean of re-admission cost in control group was about US $2751 (95% CI: 2708 – 2794) more than the intervention group (Figure 2)

**Table 2:** Meta-analysis of Re-admission Cost in Both Intervention and Control Groups.

**Meta-analysis**	**Methods**	**Pooled Estimation**	**95% CI**		*** P *** ** value**
			**Lower**	**Upper**	
Mean difference re-admission cost per patient in control group comparing to intervention group	Fixed	2750.844	2708.127	2793.560	0.000
The total saving cost per patient	Fixed	2046.64	2003.919	2089.35	0.000

**Figure 3:**
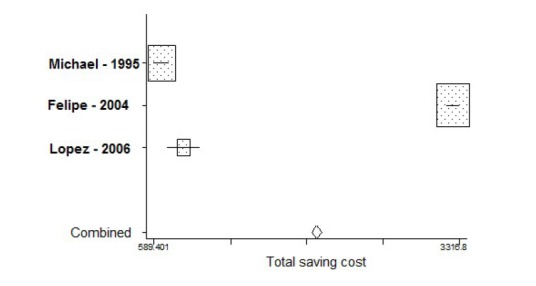
The mean of total saving cost of re-admission by written information intervention

The total saving cost in intervention group in comparison with the control group was about US $2047 (base year 2010) (95% CI: 2004 – 2089) (Figure 3)

Mean cost of re-admission in control group was about $2751 more than the intervention. Even after mincing the cost of intervention (Table 2) from the cost of readmission again about $2041 difference between the cost of readmission in control and intervention groups was observed as $2041 was saved in the intervention group. It shows that written information intervention was effective in reducing the cost of re-admission (Figure 3).

## Discussion

In the last several years, especially since 2000, minimizing the healthcare costs has been a serious issue for the governments and public.^11^ Increased use of health care, especially expensive new medical technologies, by all age groups^14^ is one of driven factors in rising the cost of health care. Patient re-hospitalization, redo procedures and operations would be other remarkable factors. People and policy makers requiring changes,^11^ and exploring evidence-based approach for this change is still open although AHRQ-funded research has found that some approaches (specific employer contribution methods, competition among health maintenance organizations [HMOs], and behavioral management managed care) save money, and others (cost sharing, flexible spending accounts, and hospital mergers) have mixed results.^11^ We believe empowering patients with relevant information would be an effective approach in minimizing re-hospitalization rate and costs, if patients know how to manage self-care. We searched for systematic reviews and meta-analysis as evidence backbone for this assumption. There were diverse studies examining the effect of different treatment approaches on the reduction of readmission but no evidence was found to show the effectiveness of written information intervention on the re-hospitalization rate and cost. Previous systematic and non-systematic reviews on the efficacy of information prescription versus oral information intervention found only disparate studies suggesting the patients’ satisfaction with information and its capability in transferring knowledge to patients.^15,16^ All the earlier reviews concluded that the evidence pool is lacking rigorous RCTs on the health outcome measures. There was no systematic review or meta-analysis with the objective of measuring health economic outcomes of written information (information prescription) intervention on hospital readmission cost. The study of López Cabezas et al^12^ found that education-based program is effective in hospital stay and “the costs derived from greater use of health resources in the control group were greater than the costs derived from the educational intervention”. Although the sample size of Atienza’s^17^ study was smaller in comparison with the other two studies, the estimated cost for intervention group in his study was lower than mean. (Figure 2).The study recruited 338 patients and found that information intervention can reduce the rate of readmission and the cost of hospitalization €2063 per patient. Rich showed that the overall cost of care was higher in the control group with an average of $153 per patient per month (Table 1).^18^ All solitary evidence was each from different countries and currency. We converted them to single monetary base of the US $ based on World Bank list and in the bottom line our meta-analysis showed that the intervention of written information (prescribed information) to specific patients leads to reduction in saving the cost of hospitalization about US $2047 per patient.

## Conclusion

Based on the systematic review and meta-analysis in this study, it is concluded that one of the effective factors in minimizing the healthcare cost and preventing from hospital re-admission is providing the patients with information prescription in a written format. It is suggested that hospital management, Medicare organizations, policy makers and individual physicians consider the prescription of appropriate medical information as the indispensable part of patient’s care process. Health information databases with patient-centered perspective need to be prepared in all nations’ native and local language in addition to international English language databases like Medline Plus, freely accessible for patients to book their doctor prescribed information and also for physicians to prescribe the information free from any commercial bias, as per information prescription standards. The clinical guidelines need to be assessed for accreditation by having included the information prescription commitment.

## Ethical issues

Not applicable.

## Competing interests

Authors declare no conflict of interests in this study.

## Acknowledgements

This study was founded by Tabriz Health Services Management Research, Center National Public Health Management Center (NPMC), Tabriz University of Medical Sciences.
